# Assessing statistical differences between parameters estimates in Partial Least Squares path modeling

**DOI:** 10.1007/s11135-016-0400-8

**Published:** 2016-08-27

**Authors:** Macario Rodríguez-Entrena, Florian Schuberth, Carsten Gelhard

**Affiliations:** 1 Department of Agricultural Economics and Rural Studies, IFAPA - Andalusian Institute of Agricultural Research and Training, Centro Alameda del Obispo, Avda. Menéndez Pidal s/n, 3092 - 14080 Córdoba, Spain; 2 Faculty of Business Management and Economics, University of Würzburg, Sanderring 2, 97070 Würzburg, Germany; 3 Faculty of Engineering Technology, University of Twente, P.O. Box 217, 7500 AE Enschede, The Netherlands

**Keywords:** Testing parameter difference, Bootstrap, Confidence interval, Practitioner’s guide, Statistical misconception, Consistent partial least squares

## Abstract

Structural equation modeling using partial least squares (PLS-SEM) has become a main-stream modeling approach in various disciplines. Nevertheless, prior literature still lacks a practical guidance on how to properly test for differences between parameter estimates. Whereas existing techniques such as parametric and non-parametric approaches in PLS multi-group analysis solely allow to assess differences between parameters that are estimated for different subpopulations, the study at hand introduces a technique that allows to also assess whether two parameter estimates that are derived from the same sample are statistically different. To illustrate this advancement to PLS-SEM, we particularly refer to a reduced version of the well-established technology acceptance model.

## Introduction

Structural equation modeling (SEM) has become a main-stream modeling approach in various disciplines, such as marketing, information systems, and innovation management (Hair et al. 2013; Henseler et al. 2014). Its ability to model complex relationships between latent constructs, to configure associations between indicators and constructs, and to account for various forms of measurement errors makes SEM a powerful statistical method for a variety of research questions. Among various approaches to SEM, including variance- and covariance-based estimators, the partial least squares path modeling (PLS) approach (Wold 1982) has particularly gained increasing attention in the last decades (Hair et al. 2014). Representing a two-step approach, PLS firstly creates proxies for the latent constructs and subsequently estimates model parameters. Since PLS is based on separate OLS regressions, no distributional assumptions are imposed on the data (’soft modeling approach’) and complex models can be estimated using a relatively small number of observations compared to the number of indicators and constructs (Henseler 2010).

Since any research method only leverages its strengths if it is properly applied in the specific research context, scholars incessantly study the limitations of PLS (Sarstedt et al. 2014; Hair et al. 2013). In so doing, scholars steadily advance PLS to broaden its applicability as well as reinforce its methodological foundations. The latest advancements to PLS refer to (i) a bootstrap-based test for evaluating the overall model fit (Dijkstra and Henseler 2015b), (ii) the heterotrait-monotrait ratio of common factor correlations as a new criterion for discriminant validity (Henseler et al. 2015), and (iii) consistent partial least squares (PLSc) as an extension of PLS, which allows for the consistent estimation of common factor and composite models (Dijkstra and Henseler 2015a). The ability to model latent constructs as both composites and common factors makes PLSc an outstanding and appealing estimator for SEM. Thus, in its most modern appearance PLS can be understood as a full-fledged SEM method^1^ which enables the hybridization of two complementary paradigm of analysis—behavioral and design research. However, PLS is still continuously enhanced. Particularly, PLS-users very often struggle with issues that are of greater practical relevance and have not been sufficiently addressed yet. One of those issues is the lack of appropriate guidance and techniques that are necessary for exploring and interpreting statistical differences between various parameter estimates (e.g., Doreen 2009 in the SmartPLS internet forum). By exploring the existence of significant differences between various parameter estimates, scholars become enabled to deepen the knowledge of both the structural model (e.g., ranking different management instruments) as well as the measurement model (e.g., identifying outstanding indicators). Commonly used practices, such as ranking various indicators/constructs based on differences in the p-values of weight/loading/path coefficient estimates or deriving conclusions solely based on effect size differences, though are prone to misleading findings and misinterpretations (e.g., Kline 2004; Vandenberg 2009; Nieuwenhuis et al. 2011; Hubbard and Lindsay 2008; Schochet 2008; Gross 2015). Gelman and Stern (2006, p. 328), for instance, accentuate that ’large changes in significance levels can correspond to small, not significant changes in the underlying quantities’. Hence, drawing conclusion about parameter differences solely based on differing p-values has to be regarded with caution, since the difference between significant and non-significant does not necessarily have to be significant (Gelman and Stern 2006).

**Fig. 1:**
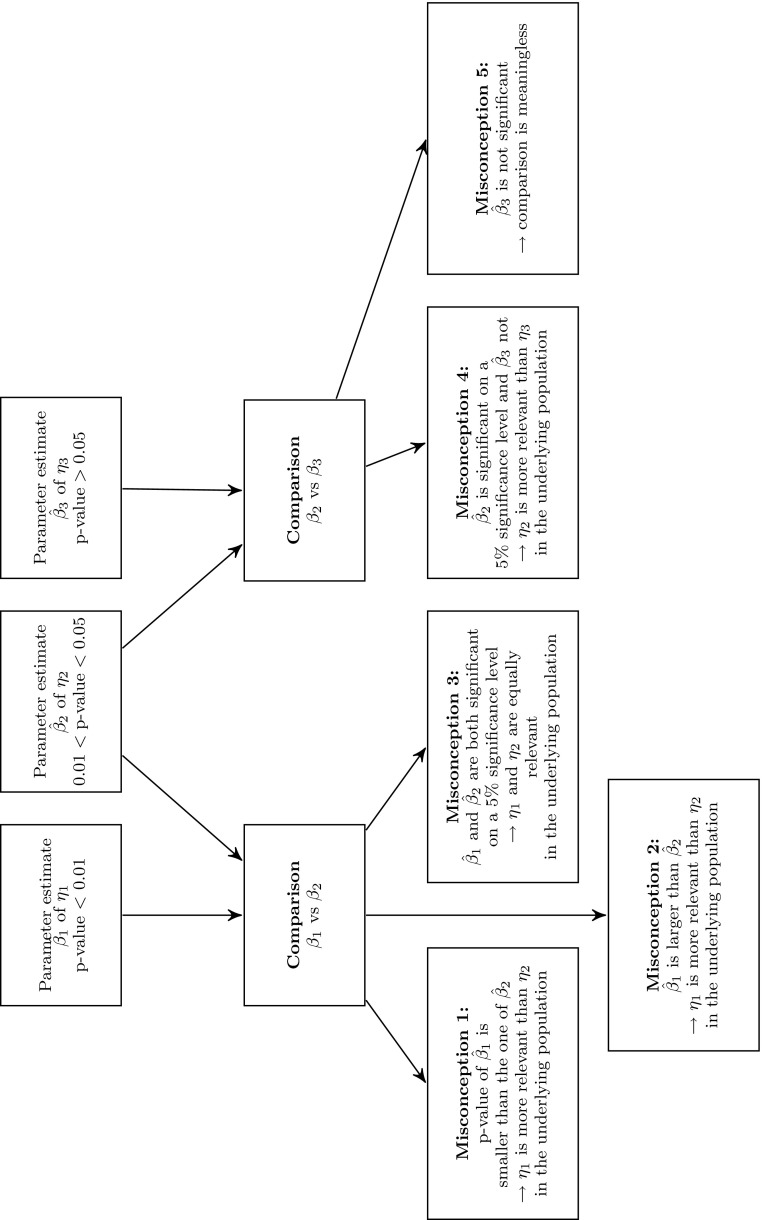
Common misconceptions in testing parameter differences

A comparison of two estimated effects rather requires a statistical test that is based on the difference between two parameter estimates rather than two separate tests for each parameter estimate. Since the mere presence of differences in p-values does not allow to make any inferences about the nature of these differences, more sophisticated steps need to be taken to fully exploit the information inherent in the SEM. Otherwise, important parameter differences might remain undetected (Gelman and Stern 2006). Figure 1 provides an overview of common misconceptions by exemplary comparing three variables ($\eta _1$, $\eta _2$, and $\eta _3$) and their related estimated coefficients ($\hat{\beta }_1$, $\hat{\beta }_2$, and $\hat{\beta }_3$, where $\hat{\beta }_1>\hat{\beta }_2$).

To eliminate these sources of misinterpretation and support PLS-users in fully leveraging information inherent in the underlying dataset, the study at hand introduces a practical guideline on how to statistically assess a parameter difference in SEM using PLS. For assessing the statistical significance of a difference between two parameter estimates, we use several bootstrap techniques which are commonly applied to test single parameter estimates in PLS. To be more precise, we construct confidence intervals for the difference between two parameter estimates belonging to the same sample. The procedure is compiled in an user-friendly guideline for commonly used PLS software packages such as SmartPLS (Ringle et al. 2015) or ADANCO (Henseler and Dijkstra 2015). By introducing this advancement, we not only fill an important gap within existing PLS literature (McIntosh et al. 2014), but also draw attention to the commonly made mistake of relying on individual p-values when prioritizing effects (Gelman and Stern 2006).

## Field of application

While most studies solely consider the estimated net effect of various predicting variables on the outcome of interest, they usually do not test whether two parameter estimates are statistically different. This prevents researchers from fully exploiting the information captured in the estimated model. Evaluating the statistical difference between two parameter estimates might be particularly valuable when model estimates are proposed to guide decision makers in handling budget constraints (e.g., selection of marketing strategies, success factors or investment in alternative instruments of innovation, process, and product, etc.). In situations in which two management instruments coexist with both having impact on the outcome of interest, a ranking of priority based on their explanatory power supports managers in selecting the most relevant. In the following, we present some empirical examples illustrating the practical relevance of assessing whether the difference between two parameter estimates belonging to the same model (i.e., comparisons within a single sample) is statistically significant.^2^

**Fig. 2:**
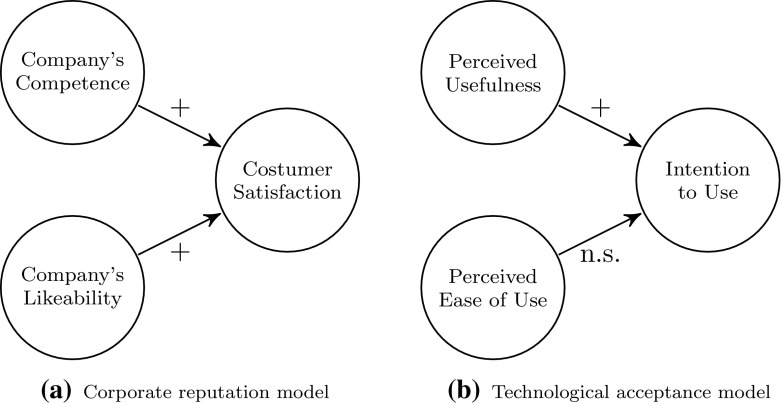
Practical examples for testing parameter differences

Figure 2a and 2b  display two excerpts of the well-known corporate reputation model (CRM) by Eberl and Schwaiger (2005) and the technological acceptance model (TAM) by Davis (1989).

Testing parameter differences might be applied to test which of the two predictors has a greater influence on the endogenous construct. To be more precise, researchers might be potentially interested in exploring whether ’Company’s Competence’ or ’Company’s Likeability’ has a higher impact on ’Customer Satisfaction’ in the context of the CRM, or, with regard to the TAM, they might be interested in statistically testing whether ’Perceived Usefulness’ is more relevant than ’Perceived Ease of Use’ in explaining ’Intention to Use’. In general, drawing conclusions solely based on the individual p-values of the estimated coefficients is not recommended (Gelman and Stern 2006) as p-values provide no information about the substantiality of a variable or the magnitude of an effect. Hence, claims such as ’Perceived Usefulness’ is more relevant than ’Perceived Ease of Use’ might be misleading (see the TAM in Fig. 2b).

**Fig. 3:**
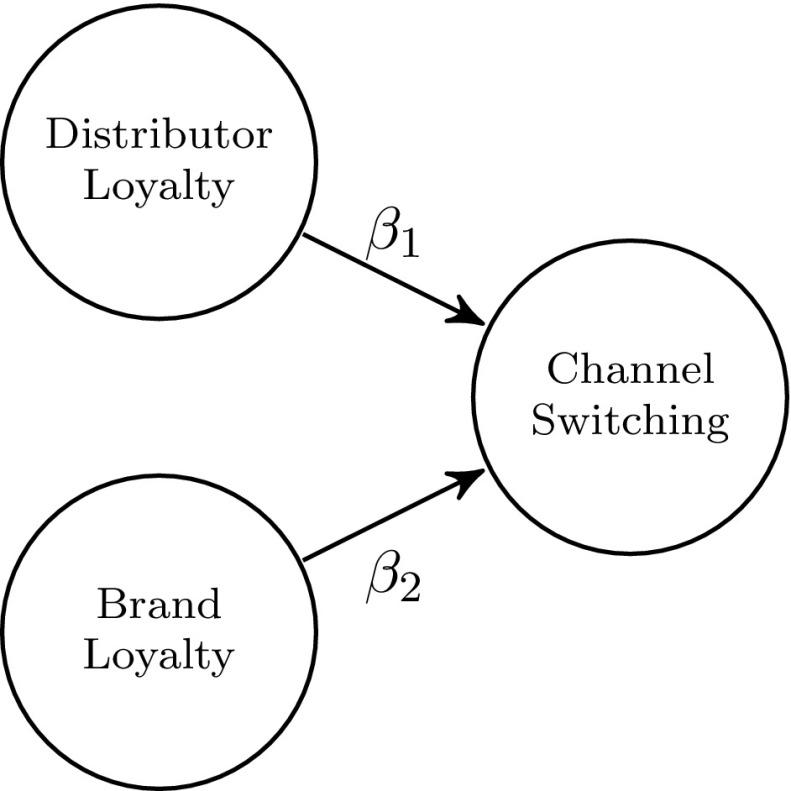
Example from Eggert et al. (2012)

In addition to the previously described examples, Fig. 3 illustrates a less common though highly interesting and important scenario: the two estimated parameters of both antecedents are approximately equal in magnitude but differ with regard to their signs ($|\hat{\beta }_1|\approx |\hat{\beta }_2|$) (Eggert et al. 2012). To eventually assess the total impact of the two antecedents on the outcome of interest (here: ’Channel Switching’) researchers might need to test whether the difference of the absolute estimated effect between both antecedents (here: ’Distributor Loyalty’ and ’Brand Loyalty’) differs significantly from zero ($H_0$: $|\beta _1| = |\beta _2|$).

## Methodological framework for testing differences between parameters

Typically in PLS, a bootstrapped based confidence interval (CI) is constructed to draw a conclusion about the population parameter. In general, a CI is designed to cover the population parameter with a confidence level $1-\alpha$. We suggest the same approach for testing a parameter difference of the following form: $\theta _k-\theta _l = 0$, see Sect. 4.^3^

In the following, we summarize the commonly used bootstrap procedures to construct CIs (Davison and Hinkley 1997) for a single parameter $\theta$ and show how these approaches can be used to assess parameter differences.^4^

### The standard/Student’s *t* confidence interval

For the standard/Student’s *t* CI it is assumed that $(\hat{\theta }-\theta )/{\widehat{{\text{Var}}({\hat{\theta}})}}^{\frac{1}{2}}$ is approximately standard normally or *t*-distributed, respectively. Since in empirical work this rarely holds, the central limit theorem is often used to justify the distribution of the standardized parameter estimates. The standard/Student’s *t* CI for a certain level of significance $\alpha$ is constructed as follows

1 $$\begin{aligned} \left[ {\hat{\theta}}-F^{-1}\left( 1-\frac{\alpha }{2}\right) \sqrt{{\widehat{{{\text{Var}}({\hat{\theta}}^*)}}}},~{\hat{\theta}}-F^{-1} \left( \frac{\alpha }{2}\right) \sqrt{{\widehat{{{\text{Var}}({\hat{\theta}}^*)}}}}\right] , \end{aligned}$$

where $\hat{\theta }$ is the parameter estimate of the original sample and $F^{-1}$ is the quantile function of the standard normal or the *t*-distribution with $n-k$ degrees of freedom, where *n* denotes the number of observations and *k* the number of estimated parameters. Since PLS does not provide an analytical closed-form of the variance, the bootstrapped-based estimator ${\widehat{{{\text{Var}}({\hat{\theta}}^*)}}}$ for the variance is used. This approach is problematic when the distribution of the parameter estimates is not normal. This is especially true for small sample sizes. Moreover, the standard/Student’s *t* CI does not adjust for skewness in the underlying population (Efron and Tibshirani 1994).

### The percentile bootstrap confidence interval

In contrast to the standard bootstrap CI, the percentile bootstrap CI is not based on distributional assumptions. The boundaries are directly calculated from the bootstrap sample distribution of the estimated parameter

2 $$\begin{aligned} \left[ \hat{F}_{\theta ^*}^{-1}\left( \frac{\alpha }{2}\right) , ~ \hat{F}_{\theta ^*}^{-1}\left( 1-\frac{\alpha }{2}\right) \right] , \end{aligned}$$

where $\hat{F}^{-1}_{\theta ^*}$ is the empirical quantile function of the bootstrap sample distribution of $\hat{\theta }$. This approach only works well if a transformation, even unknown, exists which makes the bootstrap distribution symmetric around zero (Wehrens et al. 2000). In case of no such transformation, the percentile method has to be be adjusted.^5^ However, the percentile method is really appealing due to its simplicity (Sarstedt et al. 2011).

### The basic bootstrap confidence interval

The basic bootstrap CI assumes that the distribution of $\hat{\theta }-\theta$ can be approximated by $\hat{\theta }^*-\hat{\theta }$ and therefore the quantiles of $\hat{\theta }-\theta$ are estimated by the empirical quantiles of $\hat{\theta }^*-\hat{\theta }$ (Wehrens et al. 2000). The basic bootstrap CI is constructed as follows

3 $$\begin{aligned} \left[ 2\hat{\theta }-\hat{F}_{\theta ^*}^{-1}\left( 1-\frac{\alpha }{2}\right) ;~ 2\hat{\theta }-\hat{F}_{\theta ^*}^{-1}\left( \frac{\alpha }{2}\right) \right] , \end{aligned}$$

where $\hat{\theta }$ represents the parameter estimate from the original sample, and $\hat{F}_{\theta ^*}^{-1}(1-\frac{\alpha }{2})$ and $\hat{F}^{-1}_{\theta ^*}(\frac{\alpha }{2})$ are the $1- \frac{\alpha }{2}$ and $\frac{\alpha }{2}$ quantiles of the empirical bootstrap sample distribution of $\hat{\theta }$.

## Guideline on testing parameter differences in partial least squares path modeling

Following Gelman and Stern (2006), we recommend to consider the statistical significance of the difference between two parameter estimates rather than the difference between their individual p-values when comparing two treatments. Thus, we provide a user guideline on testing a parameter difference in PLS as well as PLSc, see Table 1.

**Table 1:** Guideline for testing parameter differences based on different CI

Step 1	Use PLS or PLSc^a^ to obtain the model parameter estimates: $(\hat{\theta }_k;\hat{\theta }_l).$
Step 2	Calculate the difference of the parameter estimates: $\Delta \hat{\theta }=\hat{\theta }_k-\hat{\theta }_l.$
Step 3	Create *B* bootstrap samples of the original data set and calculate the parameter estimates $\hat{\theta }_{ki}^*$ and $\hat{\theta }_{li}^*$, and their difference $\Delta \hat{\theta }_{i}^*=\hat{\theta }_{ki}^*-\hat{\theta }_{li}^*$ for every bootstrap sample, with $i=1,...,N.$
Step 4	Estimate the variance of the estimated parameter difference as ${\widehat{{{\text{Var}}(\Delta {\hat{\theta}}^*)}}}=(B-1)^{-1}\sum \limits _{i=1}^{B}{(\Delta {\hat{\theta }}_{i}^*-\overline{\Delta{\hat{\theta}}^*})^2},\quad {\text {with}}\quad {\overline{\Delta{\hat{\theta}}^*}}=B^{-1}\sum \limits _{i=1}^{B}{\Delta{\hat{\theta}}_{i}^*}. \qquad \qquad$ (4)
Step 5	Estimate the $\frac{\alpha }{2}$ and $1-\frac{\alpha }{2}$ sample quantile of $\Delta \hat{\theta }^*$ given by $\hat{F}_{\Delta \theta ^*}^{-1}(\frac{\alpha }{2})$ and $\hat{F}_{\Delta \theta ^*}^{-1}(1-\frac{\alpha }{2}).$

^a^ PLSc should be used if constructs are modeled as common factors in the model

Firstly, the parameters of interest need to be obtained by PLS or PLSc respectively (Step 1). For this purpose, every common PLS software such as SmartPLS or ADANCO can be used. Secondly, the difference between the parameter estimates of interest is calculated (Step 2). Thirdly, the bootstrap estimates of the parameters need to be obtained (Step 3) and extracted to a spreadsheet in order to manually calculate the parameter difference for every bootstrap sample. Depending on the CI used (see Table 2), Step 4 comprises the estimation of the variance of the estimated parameter difference (e.g., VAR.S() in MS Excel). If the percentile bootstrap CI or the basic bootstrap CI is used Step 5 needs to be conducted comprising the determination of the empirical quantiles of the bootstrapped parameter difference (e.g., PERCENTILE.INC() in MS Excel).

**Table 2:** Necessary steps for the construction of the different CIs:

- Steps 1 and 2 are needed for all approaches except for the percentile bootstrap CI.	
- To apply the standard/Student’s *t* CI (Eq. 1), additionally Step 3 and 4 are necessary.	
- In contrast, the construction of the percentile bootstrap CI (Eq. 2) and the basic bootstrap CI (Eq. 3) of the parameter difference. Requires the Steps 3 and 5	

**Fig. 4:**
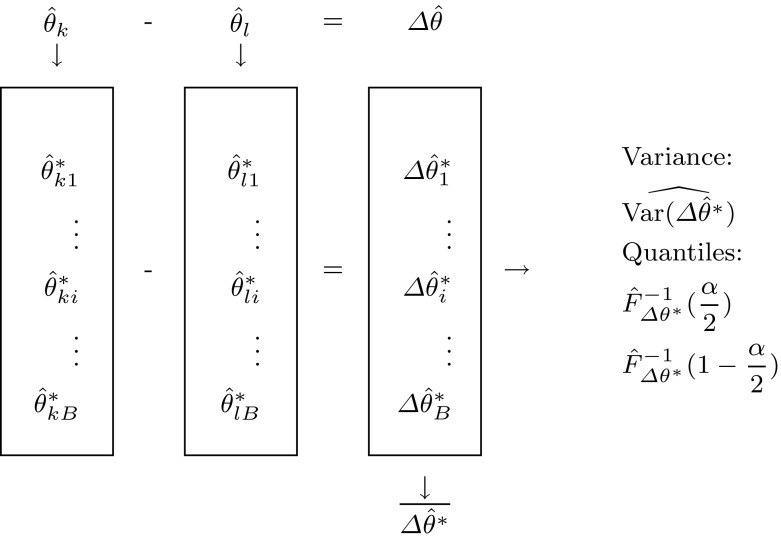
Construction of the CIs

Based on the CIs constructed the null hypothesis is rejected or not rejected. If the zero is covered by the CI, it cannot be assumed that a statistical difference between the two estimated parameters considered exists, regarding the type I error. For an illustration of the described procedure, see Fig. 4.

## Empirical example

**Fig. 5:**
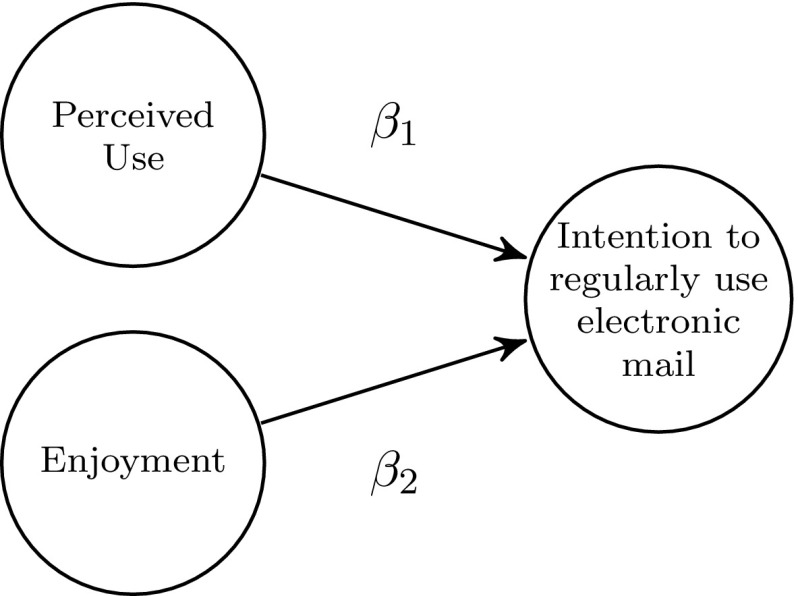
Structural model of the reduced TAM

To illustrate our proposed procedure, we refer to the TAM developed originally by Davis (1989); Davis et al. (1992), suggesting ’Perceived Usefulness’ and ’Perceived Ease of Use’ as potential predictors of IT adoption intention. More precisely, we demonstrate our procedure by referring to Chin et al. (2003), who followed Davis (1989) theoretical framework when investigating the intention to regularly use electronic mail within an organization. The data set consists of 12 indicators and 250 respondents from a single organization, which had recently installed an electronic mail system.^6^ The respondents work at different organization levels including managers, engineers, technicians, and clerical workers. The dependent construct ’Intention to regularly use electronic mail’ (INT) is explained by both ’Perceived Usefulness’ (USE) and ’Enjoyment’ (ENJ). The structural model is depicted by the following equation (see also Fig. 5):

5 $$\begin{aligned} \text {INT} = \beta _1 \cdot \text {USE} + \beta _2 \cdot \text {ENJ} + \zeta \end{aligned}$$

Following Chin et al. (2003), all constructs are modeled as common factors. While USE is measured by six indicators, both ENJ and INT are measured by three indicators each. All indicators are on a seven-point Likert scale.

Using our proposed procedure for statistically testing the difference between two parameter estimates, we seek to answer whether USE (extrinsic motivation) has a statistically different impact on INT than ENJ (intrinsic motivation) ($H_0$: $\beta _1 = \beta _2$). Since this model was originally estimated by traditional PLS but represents a common factor model, we used both approaches PLS and PLSc (Dijkstra and Henseler 2015a) for model estimation.^7^ The analysis eventually leads to the following estimated path coefficients: $\hat{\beta }_1=0.517$ and $\hat{\beta }_2=0.269$ for the model estimation with PLS and $\hat{\beta }_1=0.507$ and $\hat{\beta }_2=0.313$ for the model estimation with PLSc.

**Table 3:** Results of PLS

Type of CI (α=5 %)	Lower bound	Upper bound
Standard	0.046	0.450
Percentile	0.044	0.496
Basic	0.001	0.452

**Table 4:** Results of PLSc

Type of CI (α=5 %)	Lower bound	Upper bound
Standard	−0.099	0.488
Percentile	−0.048	0.508
Basic	−0.120	0.437

The 95 % CIs derived from the bootstrap procedure with 5000 draws (see Sect. 3) are displayed in Tables 3 and 4. Since they do not contain the zero with regard to the estimation using PLS, we infer that both path coefficient estimates ($\hat{\beta }_1$ and $\hat{\beta }_2$) are significantly different. With regard to the estimation with PLSc, all CIs cover the zero. We, therefore, conclude that the difference between the two path coefficient estimates ($\hat{\beta }_1$ and $\hat{\beta }_2$) is not statistically significant.^8^ Hence, if the underlying measurement models are conceptualized as composites (i.e., model estimation using PLS), the null hypothesis of no parameter difference ($H_0$: $\beta _1=\beta _2$) has to be rejected. If the measurement models, on the other hand, are conceptualized as common factors (i.e., model estimation with PLSc), there is not enough evidence against the null hypothesis.

## Discussion

The purpose of this paper is to provide a practical guideline as well as the technical background for assessing the statistical difference between two parameter estimates in SEM using PLS. This guideline is intended to be used to test a parameter difference based on the parameter estimates and the bootstrap distribution. The input required for the proposed methodological procedure directly builds on the output of the most popular variance-based SEM statistical software packages such as ADANCO or SmartPLS. The methodological procedure serves as functional toolbox that can be considered as a natural extension of PLS. As it is common practice in PLS to use bootstrap approaches to draw conclusions about single parameters, we use these approaches and the resulting CIs to draw conclusions about a parameter difference. As the study at hand shows, the same procedure can also be employed for PLSc to assess a parameter difference in models where constructs are modeled as common factors instead of composites.

Using the well-established TAM we eventually demonstrated the application of our proposed assessment technique. In accordance with Chin et al. (2003), we made use of PLS to test for a statistical difference between the estimated influence of ’Perceived Usefulness’ (extrinsic motivation) and ’Enjoyment’ (intrinsic motivation) on ’Intention to regularly use electronic mail’. Since no CI covered the zero, we conclude that a statistical difference between the parameter estimates exists. We also performed our proposed procedure using PLSc, since prior literature has shown that traditional PLS tend to overestimate factor loadings and underestimate path coefficients when referring to common factor models (Schneeweiss 1993). Contrasting the estimation with PLS, we cannot infer that the estimated influence of ’Perceived Usefulness’ and ’Enjoyment’ on ’Intention to regularly use electronic mail’ is statistically different. Considering the concrete example used in this study, our proposed technique has proven to be useful, i.e., when estimating the SEM using traditional PLS, we were able to show that the estimated effects of the two antecedents explaining the outcome of interest are significantly different.

Contrasting established methods for assessing whether various parameter estimates are statistically different [e.g., parametric and non-parametric approaches in PLS multi-group analysis (PLS-MGA) (Sarstedt et al. 2011)], the procedure introduced in this study enables PLS-users to test whether two parameter estimates from one sample (${\hat{\beta}}_{k}^1$ and ${\hat{\beta}}_{l}^1$) are statistically different. Approaches used in PLS-MGA, for instance, are not suitable in this framework, since the underlying assessment approach is based on the hypothesis that a parameter $\beta _k$ differs for two subpopulations (${\hat\beta}_{k}^1$ and ${\hat\beta} _{k}^2$) which can be tested, for instance, by using an unpaired *t*-test in the PLS-MGA framework (e.g., Keil et al. 2000). In the PLS-MGA framework, the proposed research model is estimated for different subsamples, followed by a comparison of the coefficient estimates across the various models. Taken together, while techniques used in PLS-MGA represent proper approaches for statistically assessing the difference between the same parameter estimate but for different subsamples ($H_0$: $\beta _{k}^i\,=\,\beta _{k}^j$, where *i* and *j* refer to the different subpopulations and *k* to the parameter tested), the procedure proposed in the study at hand represents the first choice when assessing the difference between two parameter estimates derived from the same sample ($H_0$: $\beta _{k}^i\,=\,\beta _{l}^i$, where *i* refers to the population, and *k* and *l* to the parameters tested).

Although the present study only considered path coefficient estimates while testing for differences, the proposed approach might also be performed with regard to other parameter estimates, such as weights, factor-loadings, or cross-loadings. Thus, testing for statistically significant differences between factor-loading and cross-loading estimates, for instance, might be a promising approach for evaluating discriminant validity (e.g., Hair et al. 2011; Henseler et al. 2009). Analysing whether estimated weights are significantly different might further be useful for identifying key indicators of composites. Furthermore, while the study at hand focused on explanative analysis—which still tends to be the main-stream in business research, the identification of statistical differences among parameter estimates might also become a standard procedure for predictive-analysis, which is becoming more and more pronounced in business and social science researcher (Carrión et al. 2016).

## Limitations and future research

Though we were able to introduce a diagnostic procedure for statistically assessing the differences between two parameter estimates, the study at hand is not without limitations. Firstly, we only considered the difference between one pair of parameter estimates. We, thus, recommend future research to develop procedures for testing more than two parameter estimates, following two potential approaches: (i) performing several single tests and adjust the assumed level of significance (e.g., using the Bonferroni correction) (Rice 1989), or (ii) performing a joint test, similar to a F-test in regression analysis.

Secondly, the procedure proposed in this study solely makes use of basic bootstrap approaches when calculating the required CIs. Therefore, scholars might also consider more sophisticated techniques, such as studentized, bias-corrected, tilted, balanced, ABC, antithetic, or m-out-of-n bootstrap techniques.

Thirdly, more general, scholars might in more detail investigate the performance and limitations of the various bootstrap procedures when using PLS and PLSc, in particular for small sample sizes, i.e., by a simulation study.

## Footnotes

1 For more detailed information on the state of the art of PLS please refer to Henseler et al. (2016).

2 For an overview of techniques for assessing statistical significance of differences between parameter estimatess in a multi-group setting, i.e., comparing the estimated coefficients across different sub-models, please refer to Sarstedt et al. (2011) or Henseler (2012).

3 Using some slight modifications, hypotheses of the form $\theta _k-\theta _l \ge a$ can be also tested, where $a$ is a constant.

4 We refer to Davison and Hinkley (1997) for further bootstrap procedures which overcome some limitations of the approaches presented here.

5 A well-known approach to achieve the adjustment is the bias corrected (BC) estimator (Efron and Tibshirani 1994) that is not discussed in this paper.

6 For a detailed description of the indicators, please refer to Chin et al. (2003).

7 As outer weighting scheme we used *mode A* and the *factorial* scheme was used as inner weighting scheme.

8 As PLSc path coefficient estimates are known to have a larger standard deviation compared to PLS estimates (Dijkstra and Henseler 2015a), it is not surprising that PLSc produced larger CIs than PLS.
